# A Rare Cause of Small Bowel Obstruction: A Case Report

**DOI:** 10.3389/fsurg.2022.855904

**Published:** 2022-04-26

**Authors:** Piaopiao Chen, Qiang Hu, Jinfeng Wu, Yuanshui Sun

**Affiliations:** ^1^Graduate School, Zhejiang Chinese Medical University, Hangzhou, China; ^2^Department of General Surgery, Tongde Hospital of Zhejiang Province, Hangzhou, China

**Keywords:** small bowel obstruction, bezoar, phytobezoar, surgery, case report

## Abstract

**Introduction:**

Small bowel obstruction is a common surgical emergency abdominal condition in clinical practice. Fecalith is one of the rare causative factors, especially phytobezoars.

**Case Report:**

We report the case of a 66-year-old man admitted with “abdominal pain with vomiting for 1 day.” Enhanced CT of the abdomen suggested incomplete small bowel obstruction. The symptomatic treatment with fasting, fluid replacement, gastrointestinal decompression, and antibiotics was conducted after the patient was admitted to the hospital. After 2 days of treatment, the patient's abdominal pain was not significantly relieved, so a decision was made to perform laparoscopic examination surgery. During surgery, a columnar foreign body was found embedded in the lumen of the small intestine about 10 cm away from the ileocecal region. Combined with the patient's preoperative history of consuming a large number of persimmons, the primary diagnosis of small intestinal fecalith obstruction was considered. We performed an enterotomy to remove the foreign body, and the procedure was uneventful. On postoperative day 7, the patient was successfully discharged.

**Conclusion:**

When we encounter a patient with intestinal obstruction without a history of surgery in our clinical work, we should take a careful history, especially about the consumption of foods that can cause phytoliths. When a patient has consumed a large amount of food that can cause phytobezoars before the abdominal pain, we should diagnostically consider it as phytobezoars intestinal obstruction, which helps to reduce the incidence of misdiagnosis and allows the patient to receive treatment timely and effectively.

## Introduction

Small bowel obstruction is a common surgical emergency, and phytobezoars are one of the rare causes. Phytobezoars are usually gel-like intestinal contents formed by the interaction of indigestible plant fiber, fruits, and vegetables containing acidum tannicum or pectin and gastric acid, and it can form large phytobezoars with other food residues (1). The typical clinical outcomes of intestinal obstruction will occur when this type of phytobezoars is incarcerated in a segment of the small intestine.

## Case Presentation

**Figure 1:**
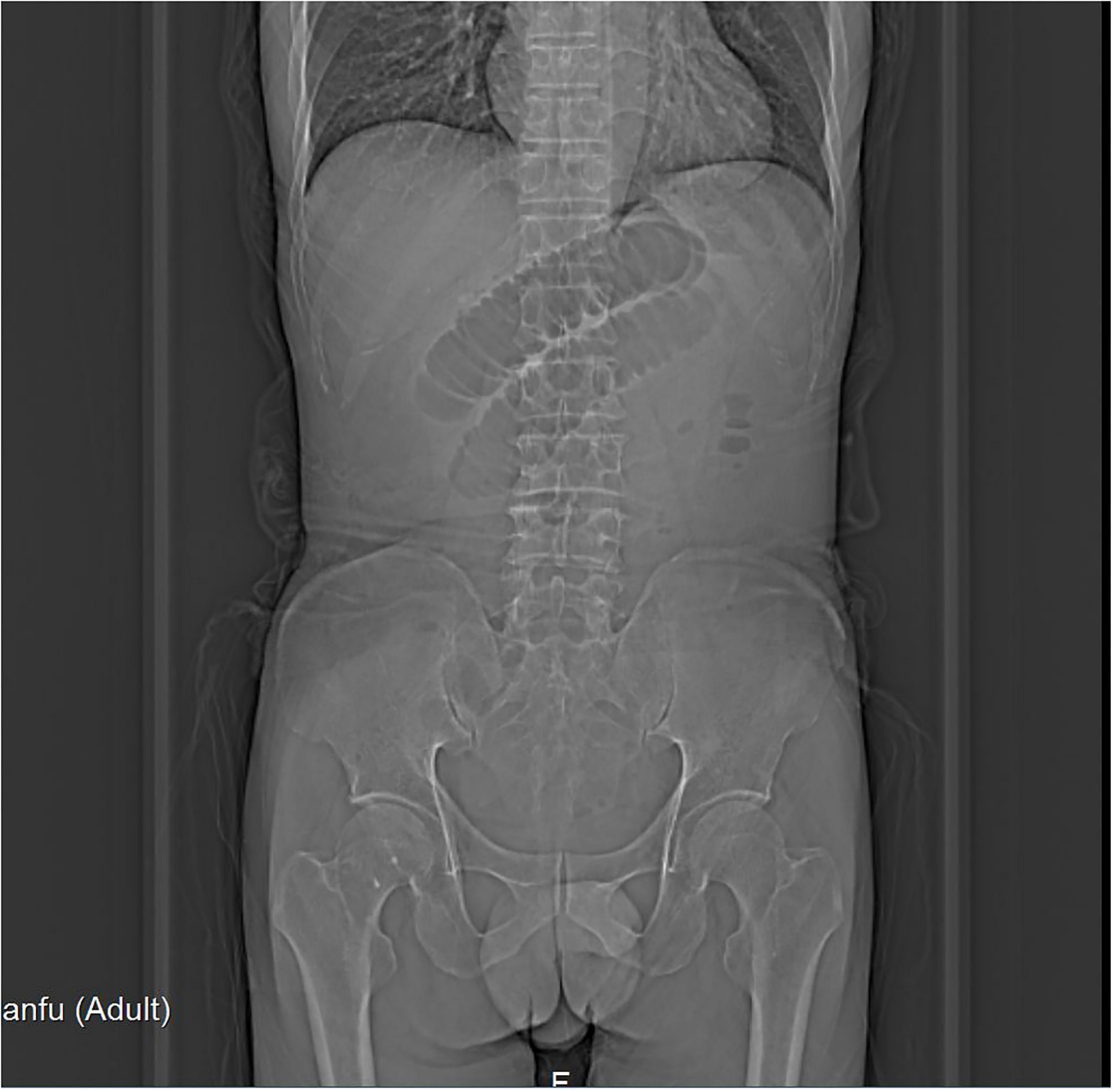
Abdominal X-ray showed intestinal obstruction.

**Figure 2:**
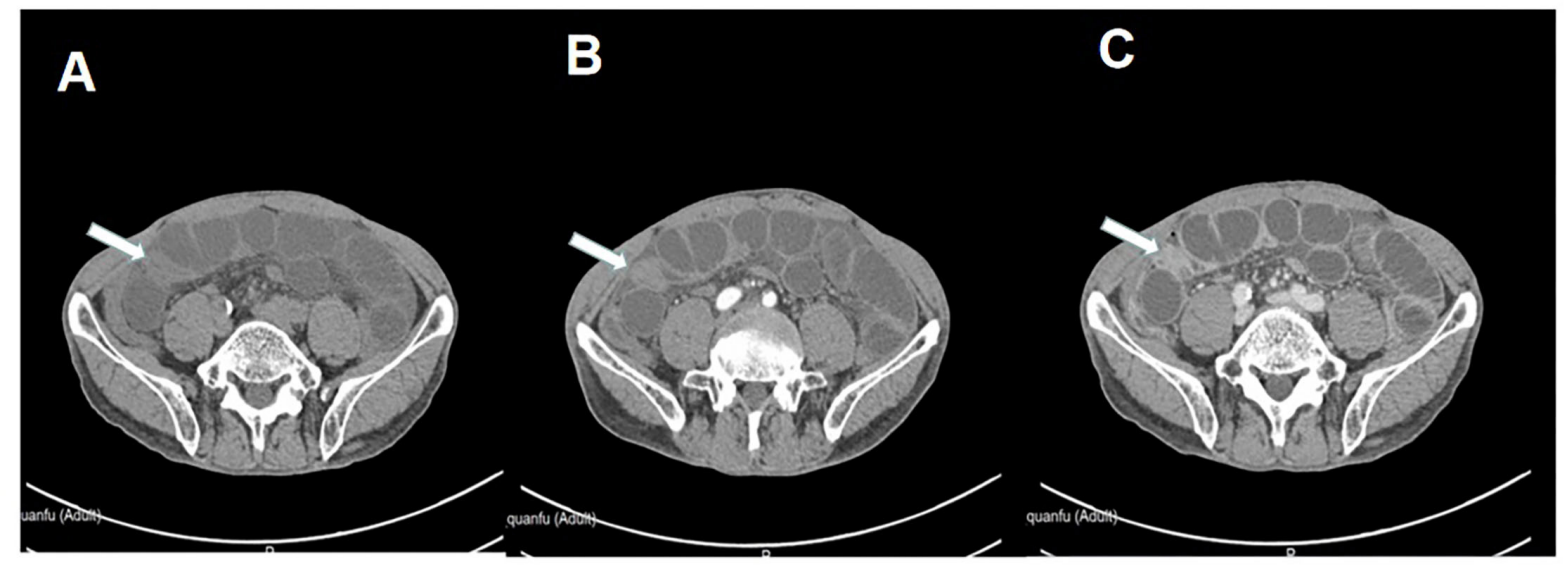
Abdominal enhanced CT suggests that incomplete small bowel obstruction should be considered, the arrow points to the site of the obstruction. **(A)** Plain scan. **(B)** Arterial phase. **(C)** Venous phase.

A 66-year-old male was admitted to the hospital with abdominal pain with vomiting for 1 day. The patient described that he had fitful abdominal pain with nausea and vomiting after a large consumption of persimmons 1 day ago. The vomit contained gastric contents. In addition, he had a failure to pass flatus and bowel movements. Physical examination found the following: temperature of 36.8°C, pulse rate of 73 bpm, respirator rate of 18 breaths/min, blood pressure (BP) of 143/88 mmHg, presence of an abdominal bulge, periumbilical pressure pain, no significant rebound pain, active bowel sounds of 6 times/min. Laboratory tests produced the following findings: white blood cell count of 10.3^*^10E9/L, neutrophil (%) of 81.6%, hemoglobin of 164 g/L, hypersensitive C-reactive protein of 11.8 mg/L. Imaging found a X-ray of the abdomen showing intestinal obstruction (Figure 1) and an enhanced CT of the abdomen showing incomplete small bowel obstruction (Figure 2). Past history was unremarkable. The patient was healthy in the past.

**Figure 3:**
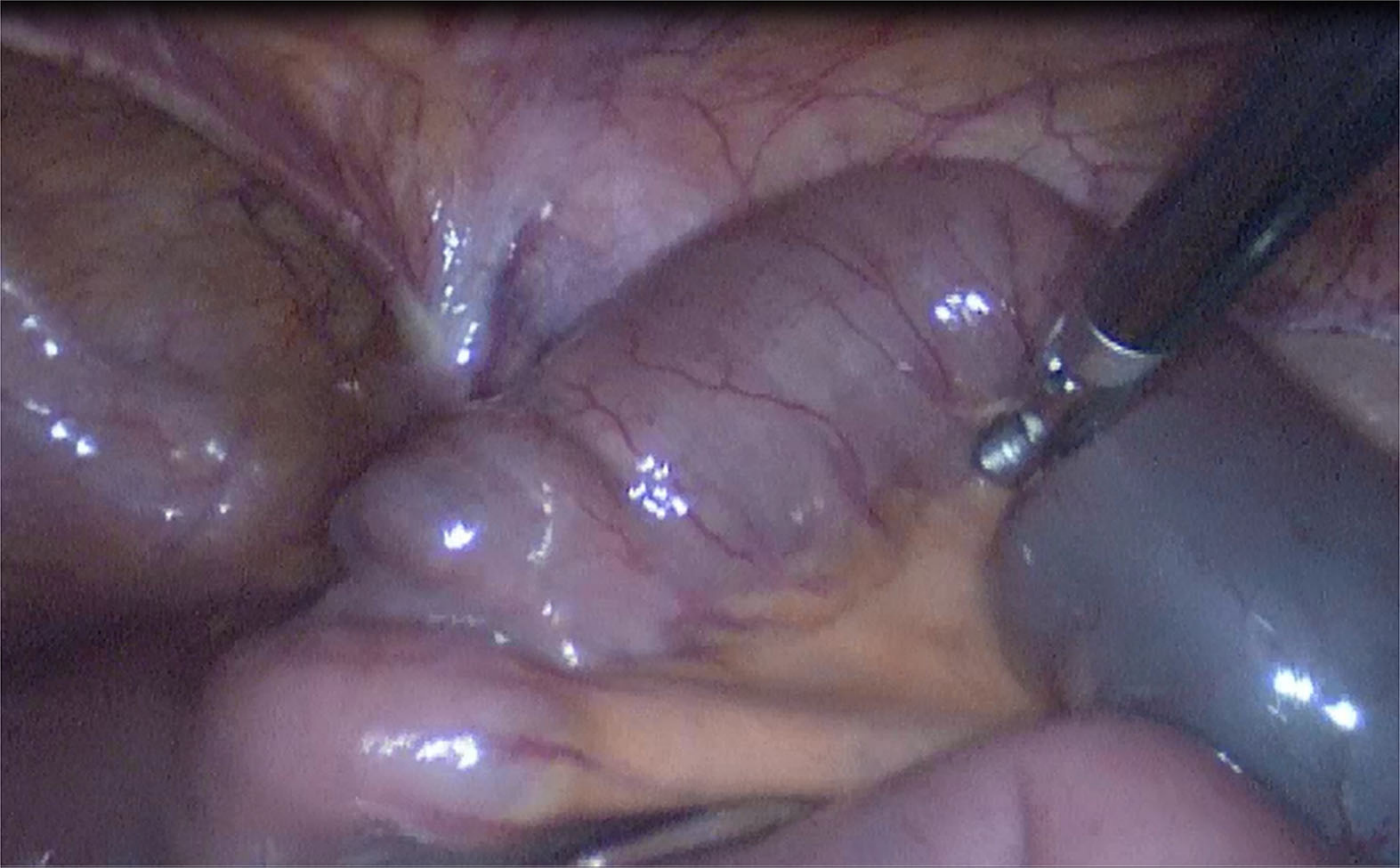
A columnar foreign body embedded in the small intestine lumen about 10 cm away from the ileocecal region.

**Figure 4:**
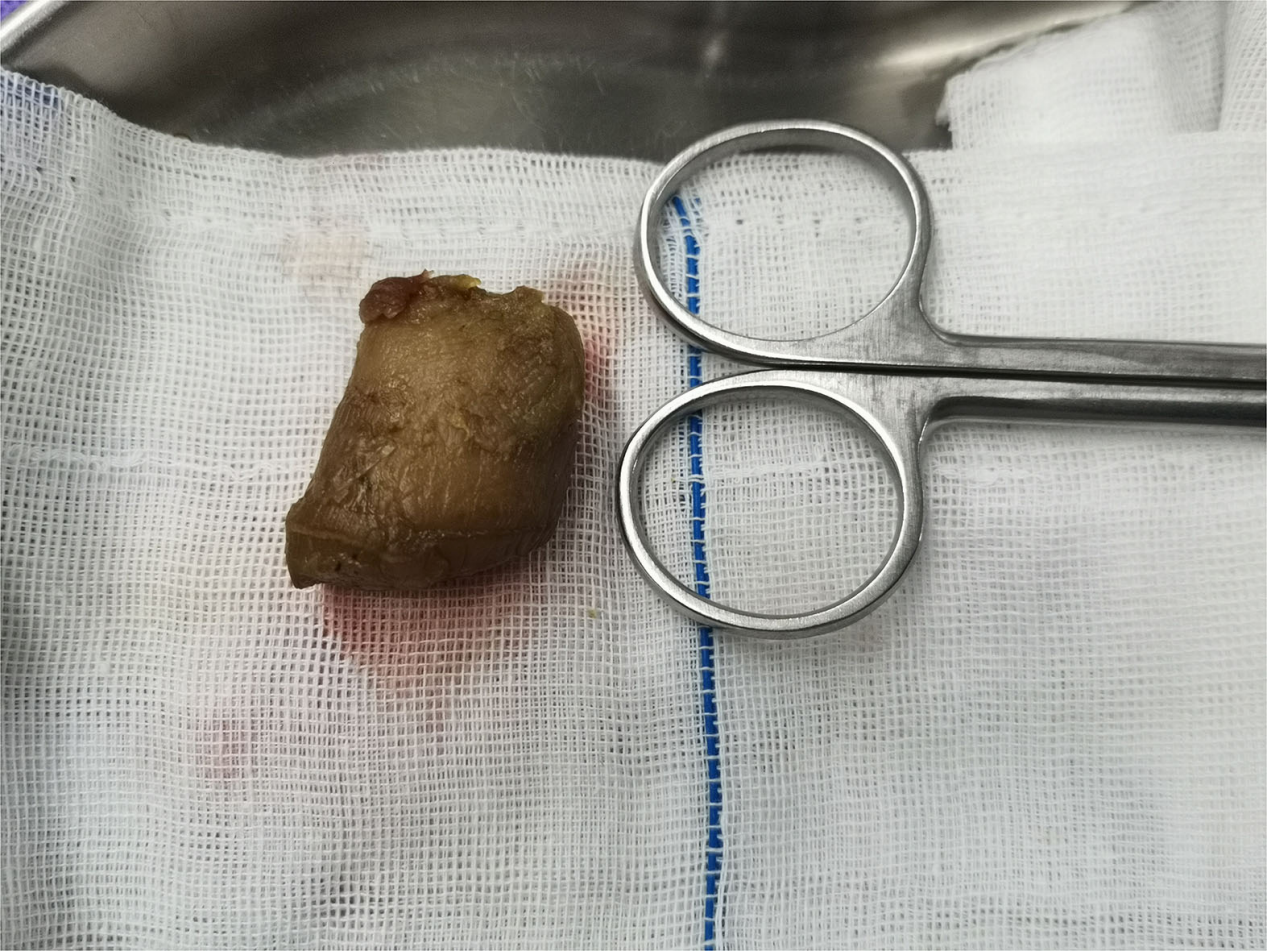
Phytobezoar is cylindrical and hard.

After admission, fasting, rehydration, gastrointestinal decompression, and symptomatic treatment with antibiotics was conducted. After 2 days of treatment, the patient's abdominal pain was not significantly relieved, and it was decided that laparoscopic exploratory surgery was to be performed. Surgery revealed a columnar foreign body embedded in the small intestine lumen about 10 cm away from the ileocecal region (Figure 3). The obstructed proximal small intestine was highly dilated, and the intestinal lumen had more contents. The distal part of the ileum and cecum of the obstruction was hollow and collapsed. The primary diagnosis is phytobezoar small bowel obstruction. Therefore, a McBurney incision was made, which was about 3 cm long and dissected into the abdomen layer by layer. The small intestine with phytobezoar incarceration was dislodged outside the abdominal incision. We made a longitudinal incision of about 4 cm above the foreign body, and the small intestine was incised. The phytobezoars were visible, hard, and cylindrical, about 2.5 cm in diameter and 3 cm length (Figure 4). The foreign body was removed, and the incision was horizontally closed with 3-0 absorbable thread. The procedure was successful. The patient had the passage of gas by anus within 2 days after the operation and was given a liquid diet. The patient also had anal bowel movements and was given a semi-liquid diet within 3 days after the operation. The patient was given a normal diet within 5 days, and laboratory tests showed a white blood cell count of 3.5^*^10E9/L, neutrophil count (%) of 55.3%, hemoglobin of 124 g/L, and ultrasensitive C-reactive protein of 5.4 mg/L. The patient made a good postoperative recovery without complications. Since the patient requested to be discharged only after the stitches were removed from the abdominal incision, the patient was not discharged until the stitches were removed from the incision on the 7th postoperative day.

## Discussion

Clinically, small bowel obstruction due to phytobezoars is uncommon, and there is no exact and available incidence data. Some literature suggests a prevalence of about 0.48%, while others suggest about 2–4% (2–4). The cause of phytobezoars-induced obstruction is mostly thought to be related to the consumption of phytobezoars-prone foods, such as persimmons, hawthorns, and sweet potatoes. On the basis of the source, the formed fecalith can be divided into phytobezoars, trichobezoar (swallowed human or animal hair), milk-based fecalith, and drug-based fecalith (barium meal), among which phytobezoars are the most common. Because of the high content of acidum tannicum and fiber in the ingested food, it can form phytobezoars with other substances under the action of gastric acid. Most cases of small intestine phytobezoars obstruction is caused by migration of gastric stones to the small intestine, and the consequential occurrence of fecalith impaction. For this patient, phytobezoar was confirmed during the surgery, and the patient confirmed that he had consumed large amounts of persimmons in his history.

Complications of patients are also an important cause of fecalith obstruction. It was found that the incidence of fecalith obstruction is about 5–12% in patients with gastric surgery, especially in patients with major distal gastrectomy, poor function of remnant gastric, and the absence of the pylorus wherein undigested food often enters the small intestine. The incidence of fecalith is about 5 to 12% (5). Diabetes mellitus is one of the predisposing factors for this disease. The presence of autonomic neuropathy in diabetic patients and gastrointestinal light paralysis in severe cases increases the retention time of food in the stomach and intestines. If these foods are consumed, they are very likely to form fecalith. It has also been found that the cause of fecalith formation is also associated with Meckel's diverticulum (6).

Fecalith small bowel obstruction is rare in clinical practice, and due to the lack of typical clinical symptoms and signs, it is easy to be missed and misdiagnosed. The size of fecalith is often small at first, but as the intestinal tract moves, the stones move downward, during which cellulose and other intestinal contents attach, gradually increasing its size. Due to the anatomical characteristics of the small intestine, the more distant the lumen of the small intestine below the treitz angle, the more difficult it is to move the fecalith to the terminal ileum. Hence, it is eventually embedded in the terminal ileum. Clinically, the symptoms of intestinal fecalith obstruction are mild in the early stage. The abdominal distension is not obvious, and it shows incomplete intestinal obstruction. As the fecalith moves to the terminal ileum, the degree of intestinal obstruction increases. Finally, it shows complete intestinal obstruction (7). Early diagnosis of fecalith intestinal obstruction is difficult, and in order to improve the correct diagnosis rate, the following points should be noted (8, 9): (1) detailed information about the presence of lithogenic foods, such as persimmons, before the onset of the disease, as it is of great help in the diagnosis of fecaliths and their causes; (2) The disease should be highly suspected in incomplete intestinal obstruction with mild initial abdominal pain, insignificant abdominal distension, mildly hyperactive or normal bowel sounds, relatively long-lasting disease, no previous surgical history, and no usual change in stool habits; (3) Abdominal X-ray examination is the basic method to diagnose intestinal obstruction, but the sensitivity of diagnosing small intestinal obstruction is low, and the cause and location of obstruction cannot be accurately determined, so the clinical application value for diagnosing the cause of obstruction is low; (4) Fecalith has a more specific performance in B ultrasound examination, but it is more difficult to locate the location of fecalith of intestinal obstruction due to the interference of gas in the intestinal cavity, so the clinical use and reference value are not better than CT; and (5), CT examination is the main examination modality for the diagnosis of this disease, and abdominal CT has important clinical value for the localization and qualitative diagnosis of fecalith intestinal obstruction. The CT diagnostic accuracy of this disease is 89.94%, and the typical CT signs include dilated and empty intestinal migratory luminal fecal ball sign with CT values of 40–80 Hu and air-containing dense spots within the mass. On the other hand, enhanced scan shows edema and thickening of the intestinal wall of the obstructed intestinal segment, in which the mass in the intestinal lumen is not enhanced (10, 11).

The treatment of fecalith small bowel obstruction should be firstly tried under close observation with conservative treatment, such as fasting, fluid replacement, gastrointestinal decompression, oral paraffin oil, and enema, as some patients can get relief. For patients who cannot be relieved, surgical exploration should be actively performed, and laparoscopic exploration is the preferred surgical method. Open surgery has deficiencies, such as more trauma, many postoperative complications, and long recovery time. At the same time, open exploratory surgery also has the disadvantages of blindness and large exploration area. The diagnosis and treatment of fecalith small bowel obstruction by the laparoscopic-assisted method have obvious advantages compared with traditional open exploratory surgery (12), namely, (1) small trauma; (2) significantly shorter recovery time of gastrointestinal function; and (3) it can effectively reduce the incidence of complications such as incisional infection, intestinal leakage, and anastomotic leakage.

Due to the anatomical characteristics of the long and tortuous small intestine, the diagnosis of the etiology solely by traditional means, such as imaging and small bowel microscopy, is easy to be missed and misdiagnosed. Laparoscopy can examine the small intestine under direct vision, which can improve the diagnosis rate of small bowel obstruction of unknown etiology by approximately 80%. It has great significance for the diagnosis of small bowel obstruction (13). (1) Laparoscopy can fully explore all the small intestine between the treiz angle and the cecum from a view outside the small intestine lumen, thus compensating for the blind spots and deficiencies of conventional imaging and small intestinal microscopy. In addition, (2) Laparoscopy can perform direct surgery for treatment while making a definite diagnosis, which is an advantage not possible with all endoscopic and imaging diagnostic methods.

Based on the location, number, size, and hardness of the fecalith, different ways of releasing the obstruction should be adopted. When the disease is long, the edema of the intestinal wall is obvious, and the stones are hard. Hence, blindly performing compression lithotripsy may easily damage the intestinal wall, or the obstruction may be formed again because the fragmented stones cannot pass through the ileocecal region. Squeeze lithotripsy is only suitable for softer fecaliths, and all fecaliths should be advanced into the colon during the procedure (14). Enterotomy is recommended for hard fecaliths. In this case, we performed a laparoscopic-assisted small bowel resection to remove the fecalith because of its hardness.

## Conclusions

When we encounter a patient with intestinal obstruction without a history of surgery in our clinical work, we should carefully inquire about the medical history, especially about the consumption of foods that can cause phytobezoars. When a patient has consumed a large amount of food that can cause phytobezoars before the abdominal pain, we should consider the diagnosis of fecalith intestinal obstruction, which can help reduce the incidence of misdiagnosis and thus enable the patient to receive timely and effective treatment.

## Data Availability Statement

The original contributions presented in the study are included in the article/Supplementary Material, further inquiries can be directed to the corresponding author/s.

## Ethics Statement

The studies involving human participants were reviewed and approved by Tongde Hospital of Zhejiang Province. The patients/participants provided their written informed consent to participate in this study. Written informed consent was obtained from the individual(s) for the publication of any potentially identifiable images or data included in this article.

## Author Contributions

QH and PC designed this study and wrote the manuscript. JW and YS collected the information and images. YS reviewed the manuscript. All authors read and approved the final manuscript.

## Funding

This study was supported by the Science and Technology Planning Project of Zhejiang Province (No. 2017F30045), Science and Technology Planning Project of Traditional Chinese Medicine (No. 2018ZZ004), and Gastrointestinal surgery of integrated traditional Chinese and Western Medicine (No. 2017-XK-A20).

## Conflict of Interest

The authors declare that the research was conducted in the absence of any commercial or financial relationships that could be construed as a potential conflict of interest.

## Publisher's Note

All claims expressed in this article are solely those of the authors and do not necessarily represent those of their affiliated organizations, or those of the publisher, the editors and the reviewers. Any product that may be evaluated in this article, or claim that may be made by its manufacturer, is not guaranteed or endorsed by the publisher.
